# Qualitative Histological Evaluation of Various Decalcifying Agents on Human Dental Tissue

**DOI:** 10.1055/s-0042-1755615

**Published:** 2022-10-11

**Authors:** Dusit Bumalee, Puangwan Lapthanasupkul, Khumpee Songkampol, Natchalee Srimaneekarn, Nakarin Kitkumthorn, Tawepong Arayapisit

**Affiliations:** 1 Department of Oral and Maxillofacial Pathology, Faculty of Dentistry, Mahidol University, Bangkok, Thailand; 2 Department of Oral and Maxillofacial Radiology, Faculty of Dentistry, Mahidol University, Bangkok, Thailand; 3 Department of Oral Biology, Faculty of Dentistry, Mahidol University, Bangkok, Thailand; 4 Department of Anatomy, Faculty of Dentistry, Mahidol University, Bangkok, Thailand

**Keywords:** decalcifying agent, dental, histology, teeth

## Abstract

**Objective** Dental hard tissue is among the hardest tissue of humans because it contains high amounts of inorganic substances. This leads to difficulty in preparing histological sections for histopathological examination. Acid and chelating agents are generally used to decalcify teeth. We aimed to compare the histological quality of teeth decalcified with various calcifying agents including 5% nitric acid, 50% formic acid with 20% sodium citrate (Anna Morse solution), 10% formic acid, commercial solution, and 14.4% neutral EDTA.

**Materials and Methods** Freshly extracted premolar teeth were fixed and submitted for decalcification using different agents. Histological examination was qualitatively evaluated for tissue integrity and staining quality.

**Results** Dentin integrity of teeth decalcified with all decalcifying agents did not show any statistical differences except that with the formic acid, whereas cementum integrity decalcified with neutral EDTA showed a superior score compared with other agents. Tissue integrity and staining quality of dental pulp cells were the best decalcified with neutral EDTA or Anna Morse solution.

**Conclusion** Our findings demonstrated that EDTA and Anna Morse solution gave a similar efficiency in the preservation of tissue integrity while Anna Morse solution may be recommended as a decalcification agent in routine use due to the more satisfying decalcification time than EDTA.

## Introduction

Histological examination of dental hard tissue and dental pulp is essential for establishing the diagnosis of pulpal diseases, developmental disorders, forensic odontology, and dental research. 1 2 3 4 To obtain thin sections, conventional tissue processing methods are not possible for the hard tissue such as bone, teeth, and calcified lesions. Decalcification, a process of removing calcium from hard tissue, is routinely conducted in all histological and histopathological laboratories to make the tissue soft enough to be cut by the microtome. 5 The goal of decalcification is to remove calcium ions from the mineralized tissue, leading to the preservation of the organic substance using various decalcifying acids or chelation agents forming a complex with calcium. 6 Strong acid takes a short time for decalcification although it damages tissue and reduces the staining quality of the tissue. In contrast, chelating agents preserve tissue structure and give better tissue stainability, but they take a longer time. However, the effect of the decalcifying agents also depends on various factors including concentration used, temperature, time duration for decalcification, tissue suspension, size and type of mineralized tissue. 1 7

The purpose of this study was to evaluate qualitative histological features of human premolar teeth using various decalcifying agents. Dental hard tissue and soft tissue including dentin, cementum, and dental pulp tissue were histologically evaluated.

## Materials and Methods

Ethical consideration of this study was reviewed and exempted by the Institutional Review Board of Faculty of the Dentistry/Faculty of Pharmacy, Mahidol University, Bangkok, Thailand (COE.No. MU-DT/PY-IRB 2018/054.1211). Sixty freshly extracted human premolar teeth that were removed for orthodontic purposes were included in the study. The tooth had to be normal in morphology and free from caries or any restorations. After extraction, they were immediately fixed in 10% neutral buffer formalin for up to 24 hours 8 and were assigned into five groups ( *n* = 12) as follows: Group 1: 5% nitric acid (Merck, Germany), Group 2: Anna Morse solution (50% formic acid [VMR, UK], with 20% sodium citrate [KemAus, Australia]), Group 3: 10% formic acid (VMR, UK), Group 4: commercial solution (Surgicpath Decalcifier II, 3800420, Leica Biosystems, IL, USA), and Group 5: 14.4% neutral EDTA (UNIVAR, Australia).

For decalcification of each group, four teeth were suspended using thread in a separate container containing 250 mL of the assigned decalcifying agent at room temperature on a magnetic stirrer. The freshly prepared decalcifying agents were renewed twice a week. The teeth were periodically checked, and the end point of decalcification was estimated by physical and radiographic methods. 1 9 The physical method was performed by bending and probing the tooth using a fine needle. When the needle passed through the whole tooth thickness, the decalcification process was then over. 10 The radiographic method was then assessed by exposing 60 KV, 8 mA for 0.1 second, and an absence of radiopacity confirmed a complete decalcification. After decalcification, the teeth were washed under tap water overnight 9 and continued for routine tissue processing in the automatic tissue processor. The specimens were dehydrated through an alcohol series from 70% to absolute. The teeth were cleared with xylene and then embedded in paraffin wax. The received blocks were sectioned on microtome for 3 µm slices, and the sections were stained with the hematoxylin and eosin method. 11 The stained section was observed under a light microscope by two examiners blinded to the groups, and histological evaluation of each dental tissue including dentin, cementum and dental pulp tissue was graded from 1 to 3 points score based on the criteria shown in Table 1 .

**Table 1:** The histological evaluation parameters and scoring criteria

Parameters	Score	Criteria
1. Dentin integrity	1	< 10% of areas showing intact pre-dentin and regular dentinal tubule pattern
	2	< 50% of areas showing intact pre-dentin and regular dentinal tubule pattern
	3	≥ 50% of areas showing intact pre-dentin and regular dentinal tubule pattern
2. Cementum integrity	1	< 10% of areas showing intact cementum layer with incremental line
	2	< 50% of areas showing intact cementum layer with incremental line
	3	≥ 50% of areas showing intact cementum layer with incremental line
3. Integrity of cells within dental pulp (odontoblasts, dental pulp cells)	1	< 10% of areas showing intact pulp tissue morphology
	2	< 50% of areas showing intact pulp tissue morphology
	3	≥ 50% of areas showing intact pulp tissue morphology
4. Staining of cells within dental pulp (odontoblast, dental pulp cell)	1	< 10% of areas showing pulpal cells showing nuclear and cytoplasmic staining
	2	< 50% of areas showing pulpal cells showing nuclear and cytoplasmic staining
	3	≥ 50% of areas showing pulpal cells showing nuclear and cytoplasmic staining

## Results

From Table 2 , the time required for complete decalcifying agents was least in 5% nitric acid while 14.4% neural EDTA required the longest time among the five groups of decalcifying agents.

**Table 2:** Estimated time for complete decalcification in each group

Groups	Decalcifying Agents	Time (days)
1	5% Nitric acid	6–7
2	50% Formic acid+ 20% sodium citrate-Anna Morse solution	8–10
3	10% Formic acid	13–15
4	Commercial decalcifier	14–18
5	14.4% Neutral ethylenediaminetetraacetic acid	45–50

**Fig. 1:**
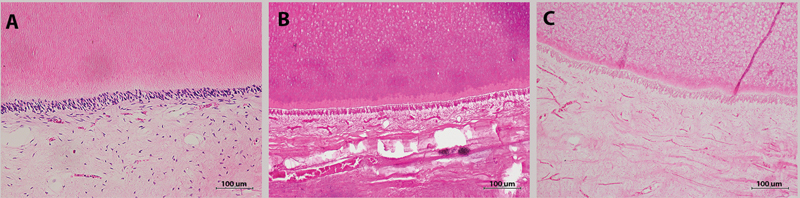
Representative histological photos of dental tissue decalcified with different decalcifying agents; EDTA ( **A** ), Anna Morse solution ( **B** ) and nitric acid ( **C** ).

Regarding the qualitative evaluation of histological features of decalcified teeth ( Table 3 ), all dental tissue including dentin, cementum, and cells within the dental pulp were determined ( Fig. 1 ). All decalcifying agents can preserve dentin with no statistical significance ( *p* > 0.05) except the formic acid, which showed less score of dentin integrity compared with other agents with statistical significance ( *p* < 0.001).

**Table 3:** Mean score and standard deviation of each histological parameter in decalcifying agents

Decalcifying agent	Dentin integrity	Cementum integrity	Integrity of cells within dental pulp (odontoblast, dental pulp cell)	Staining of cells within dental pulp (odontoblast, dental pulp cell)
Neutral EDTA	2.08 ± 0.28	2.58 ± 0.51 ^b,c,d^	1.50 ± 0.79	2.33 ± 0.98
Anna Morse	2.25 ± 0.62	1.83 ± 0.57 ^a,d^	1.83 ± 0.93	1.75 ± 0.96
Commercial solution	2.33 ± 0.50	2.11 ± 0.78 ^a,b,d^	1.22 ± 0.44	1.33 ± 0.50 ^a^
Nitric acid	2.00 ± 0.00	1.10 ± 0.31 ^a,b,c^	1.60 ± 0.84	1.20 ± 0.42 ^a^
Formic acid	1.09 ± 0.30 ^a^	1.18 ± 0.40 ^a,b,c^	1.00 ± 0.00	1.00 ± 0.00 ^a^

a *p* < 0.5 compare with Neutral EDTA group.

b *p* < 0.5 compare with Anna Morse group.

c *p* < 0.5 compare with Commercial solution group.

d *p* < 0.5 compare with Nitric group.

For the cementum, neutral EDTA was superior to other agents with statistical significance, followed by commercial solution and Anna Morse solution. Nitric acid and formic acid preserved cementum inferior to other agents with statistical significance.

With regard to pulpal cells, Anna Morse gave the highest score of pulpal integrity, but all decalcifying agents did not show any statistically significant differences ( *p* > 0.05). The quality of pulpal staining was the best seen in the teeth decalcified with neutral EDTA. The staining quality of pulpal cells decalcified with neutral EDTA and Anna Morse solution was not significantly different, though these two agents were still superior to the others with statistical significance.

## Discussion

In the present study, we compared five decalcifying agents including nitric acid, Anna Morse solution, formic acid, commercial solution, and neutral EDTA. The qualitative histological evaluation of various decalcifying agents on human teeth was compared on the following parameters: decalcification time, dentine integrity, cementum integrity, integrity of cells within the dental pulp, and staining of cells within dental pulp. Our study found that the decalcification time of nitric acid was the shortest and EDTA was the longest, in accordance with previous studies. 1 10 11 Because the solubility of calcium phosphate largely depends on solution pH, 12 EDTA with neutral pH takes a longer time compared with acid with a lower pH. Furthermore, EDTA is a chelating agent that gradually captures calcium ions from the superficial surface of apatite crystals and then slowly reduces the size of crystals. This prevents tissue collapse and preserves tissue architecture superior to acid decalcifying agents. 1

Regardless of decalcification time, our findings pointed out that EDTA was suitable for decalcification for histological examination of both dentin and cementum. Although no statistical difference was observed for the histological score of dentin among the chemicals except the formic acid, EDTA tended to preserve dentin morphology better than the others. For cementum integrity, the sample treated with EDTA showed slight cementum destruction and separation from dentin, while cementum damage was seen in other acid decalcification agents, which is in agreement with that reported in the previous study. 13 The reason behind this phenomenon might be caused by the lytic activity of acid destroying the tissue architecture, while the chelating ability of EDTA helps to prevent the collapse of tissue after the removal of the mineral element.

Microscopic analysis of pulpal tissue is of importance in studying tissue response not only in disease but also in treatment technique and treatment materials. The preservation of the pulp zone and intensity of staining indicates tissue quality. Appropriate preservation and decalcification could minimize the possible alteration during histological preparation. If the decalcification process is not complete, the pulpal tissue is vulnerable and frequently torn down during the sectioning process. In contrast, prolonged decalcification affected the integrity of dental tissue by increasing the possibility of distortion of the superficial layer of specimens that came in contact with the decalcifying solution, thus leading to decreased tissue stability. 14 15 Our results showed that the Anna Morse solution and EDTA were superior to other solutions in pulpal tissue preservation. Although no significant difference in outcome was observed between both decalcifying agents, the pulpal integrity from the teeth decalcified with the Anna Morse solution gave higher score quality than that with EDTA. A similar result was obtained in a previous study, 8 in which primary teeth with inactive carious lesion decalcified with EDTA demonstrated more alterations of soft tissue architecture than those decalcified with the Anna Morse solution. This may be explained by the prolonged period of complete decalcification of EDTA. 9 Therefore, the Anna Morse solution may be preferably selected in decalcification in the study requiring a high quality of pulpal tissue.

Anna Morse solution consists of formic acid added with sodium citrate. With regard to formic acid, it produces more notable pulpal damage after decalcification than other chemicals, consistent with previous reports. 9 10 Owing to the rapid removal of calcium ions of acid, dentinal tubules were then exposed and created a direct channel for acid to reach the pulp tissue, thus possibly damaging the odontoblastic cell layer and detaching the pulp from the dentin wall. 13 16 The number of hydrogen ions released from acid contacted the tissue surface and then provoked the alterations on both cells and extracellular matrix by compromising the antigen marker, 17 resulting in poor cationic dye staining of the nucleus and swelling of collagen fiber. 13 14 15 To counterbalance the acidity, a combination of formic acid and sodium citrate was introduced in 1930. 18 Subsequently, experiments were performed to modify the proportion of both chemicals and improve the ease of sectioning and the histological quality, becoming the so-called Anna Morse solution. 14 Because the addition of sodium citrate aimed to neutralize the acidity of formic acid, a mixture of Anna Morse solution not only exhibited rapid decalcification as a benefit of acid but also prevented soft tissue swelling with excellent nuclear staining due to the buffer capacity of sodium citrate, 8 19 thus, resulting in better tissue preservation than acid while taking shorter decalcification period than EDTA.

## Conclusion

An attempt has been made to appraise the suitable decalcification agent by balancing time factors and tissue quality. In the present study, acid decalcifying agents distorted, especially, soft tissue integrity, suggesting the use with caution in histological interpretation. Although EDTA gave an excellent result in both hard and soft tissue preservation, Anna Morse solution may be superior because it yielded an adequate tissue quality but a short time for decalcification, suggesting the use of this agent in routine histopathological diagnosis and research.
